# Association of nasal irrigation with reduced nasal symptoms after radioiodine ablation in differentiated thyroid cancer: a retrospective cohort study with propensity score matching

**DOI:** 10.3389/fendo.2026.1897010

**Published:** 2026-09-24

**Authors:** Juan Ren, Shuiyuan Fu, Yuanfei Dong, Yueyun Ding, Feibo Zheng, Shasha Meng, Zongping Zhu, Shuai Song

**Affiliations:** 1 Department of Nuclear Medicine, Qingdao Hospital, University of Health and Rehabilitation Sciences (Qingdao Municipal Hospital), Qingdao, China; 2 Qingdao Medical College of Qingdao University, Qingdao, China

**Keywords:** differentiated thyroid cancer, nasal irrigation, nasal symptoms, propensity score matching, radioactive iodine, retrospective cohort study, target-to-background ratio

## Abstract

**Background:**

Nasal symptoms after radioactive iodine (RAI) therapy in differentiated thyroid cancer have received less attention than salivary or lacrimal toxicity. Nasal saline irrigation is a simple supportive-care measure, but its association with post-RAI nasal symptom burden remains insufficiently evaluated.

**Methods:**

This single-center retrospective cohort study included patients with differentiated thyroid cancer who received ^131^I remnant ablation or therapy at Qingdao Municipal Hospital between April 2024 and March 2025. Patients received either standard care alone or standard care plus stepped nasal irrigation with 0.9% saline. Nasal symptoms were assessed using a 0–10 visual analog scale at discharge and at 2 weeks, 1 month, and 2 months after discharge. Propensity score matching was used to construct the matched cohort. Post-therapy imaging on days 3 and 7 was used to explore nasal mucosa target-to-background (T/B) ratios.

**Results:**

Among 185 eligible patients, 92 received standard care and 93 received nasal irrigation. After matching, 148 patients were included in the primary analysis. Symptoms meeting the prespecified VAS ≥3 threshold occurred in 27 of 74 patients (36.5%) in the standard-care group and 13 of 74 patients (17.6%) in the irrigation group (risk difference, −20.27 percentage points; 95% CI, −34.32 to −6.22; P = 0.005). Rescue medication was required in 25 of 74 patients (33.8%) and 11 of 74 patients (14.9%), respectively. Nasal dryness showed the most consistent between-group difference. The day-7 T/B ratio was lower in the irrigation group (2.24 ± 0.76 vs. 2.56 ± 0.81; P = 0.012), whereas the day-3 ratio was similar.

**Conclusions:**

In this retrospective cohort, stepped nasal saline irrigation was associated with fewer early post-RAI nasal symptoms and less rescue medication use. The imaging findings were exploratory and should not be interpreted as direct evidence of enhanced radioiodine clearance. Prospective studies are needed to confirm these findings.

## Introduction

Radioactive iodine (RAI, ^131^I) therapy is routinely used in the postoperative management of selected patients with differentiated thyroid cancer (DTC). Because the potential oncologic benefit must be balanced against non-thyroidal toxicity, current guidelines advocate risk-stratified administration of RAI (1, 2). Postoperative assessment with serum thyroglobulin and neck ultrasonography also remains central to risk-adapted follow-up in DTC (3). In addition to its established therapeutic value, RAI therapy is associated with a spectrum of acute and chronic adverse effects involving salivary, lacrimal, and upper aerodigestive tract tissues (4–10). These adverse effects are increasingly understood not only as organ-specific toxicities, but also as patient-reported symptom burdens that may affect comfort, adherence to supportive care, and quality of life during recovery.

RAI-related salivary gland injury has received sustained attention in the literature (11), whereas nasal adverse effects are less consistently assessed in routine thyroid cancer follow-up. Prior studies have reported nasal discomfort, dryness, obstruction, epistaxis, and olfactory changes after RAI therapy (12–16). Nasal complaints have also been discussed in relation to lacrimal complications and broader craniofacial symptom burden after RAI, including recently developed patient-reported outcome instruments such as the SAlivary, LAcrimal, and NaSal symptom measure (SALANS) (17). These symptoms may be transient and mild in many patients, yet they can emerge within days of treatment and compound the discomfort of an already demanding recovery. Because nasal symptoms are often not the primary focus of post-RAI assessment, their frequency, severity, and modifiability remain incompletely characterized.

A local-exposure mechanism may explain these symptoms. Extrathyroidal iodide transport, mediated at least in part by sodium/iodide symporter expression, leads to accumulation of radioiodine in secretions of the nasal mucosa (18, 19). Post-therapy imaging studies have further shown intranasal localization of ^131I^, particularly in patients receiving higher administered activities, supporting the biological plausibility of radioiodine-containing nasal or nasolacrimal secretions as a contributor to local toxicity (20). Prolonged contact between radioactive secretions and the nasal epithelium could promote mucosal irritation and compromise mucociliary clearance. Nasal irrigation offers a mechanistically straightforward means of reducing this local exposure by physically removing secretions and restoring mucosal hydration. Previous rhinology studies have demonstrated that saline irrigation improves mucociliary clearance, reduces secretion retention, and alleviates inflammatory sinonasal symptoms (21–27). However, whether this simple supportive-care approach is associated with fewer nasal symptoms after systemic RAI therapy has not been adequately evaluated.

Against this background, we conducted a single-center retrospective cohort study to examine whether a stepped nasal saline irrigation protocol was associated with reduced RAI-related nasal symptoms in patients with DTC. Post-therapy whole-body scintigraphy/SPECT-based imaging was used to explore whether the nasal mucosa T/B ratio provided an imaging correlate of local nasal radioiodine retention. The study was conceived as a pragmatic clinical evaluation of a low-cost nursing measure, not as a substitute for a randomized trial.

## Materials and methods

### Study design and setting

This single-center retrospective cohort study was conducted at Qingdao Municipal Hospital. The clinical data were derived from an institutional quality-improvement practice for supportive care after radioactive iodine (RAI) therapy. The institutional project period covered April 2024 to March 2025, during which consecutive patients receiving ^131^I remnant ablation or therapy were screened. The present manuscript represents a retrospective analysis of de-identified clinical, nursing, imaging, medication, and follow-up data collected during that period.

During implementation of the institutional supportive-care practice, eligible patients were managed according to admission sequence under a predefined approximately 1:1 ward-based care arrangement: standard care alone or standard care plus stepped nasal irrigation guidance. This explains why the original group sizes were similar. No intervention was newly assigned for the purpose of the present retrospective analysis. The protocol for retrospective data analysis was reviewed and approved by the Medical Ethics Committee of Qingdao Municipal Hospital (approval No. 2026-KY-077). Given the retrospective nature of the analysis and use of de-identified data, the ethics committee waived the requirement for individual written informed consent. Reporting follows the STROBE recommendations where applicable (28).

### Participants

A total of 208 patients were screened. Twenty-three patients were excluded before construction of the final retrospective cohort: four because adequate hydration could not be ensured owing to cardiopulmonary conditions, eight because of pre-existing nasal disease, two because of nasal symptoms during the treatment admission, eight because of irrigation-related discontinuation or inability to complete the prespecified irrigation protocol, and one because of loss to follow-up. The final analytic cohort therefore included 185 eligible patients.

The inclusion criteria were: (1) pathologically confirmed differentiated thyroid cancer, including papillary or follicular carcinoma; (2) total or near-total thyroidectomy before RAI therapy; (3) first or repeat ^131^I remnant ablation or therapy; (4) age 18–75 years; and (5) complete clinical and follow-up data for the relevant analysis. Patients were excluded if they had chronic rhinosinusitis, allergic rhinitis, nasal polyps, or other pre-existing nasal disease; nasal symptoms before or during the RAI treatment admission; severe cardiopulmonary, hepatic, or renal dysfunction that precluded adequate hydration or safe completion of the supportive-care protocol; pregnancy or lactation; inability to complete the assigned supportive-care protocol; or loss to follow-up.

Preparation for ^131^I therapy was performed without recombinant human thyroid-stimulating hormone (rhTSH). Patients followed a low-iodine diet and achieved endogenous TSH stimulation by withholding thyroid hormone supplementation after surgery or discontinuing thyroid hormone therapy for 2–4 weeks before ^131^I administration.

Baseline demographic and clinical variables included age, sex, pathological type, TNM stage, surgical procedure, administered ^131^I activity, number of ^131^I treatments, smoking history, and length of inpatient stay. Eligibility screening also recorded whether patients had pre-existing nasal disease, recent upper respiratory tract infection, inability to maintain the hydration protocol, or baseline nasal symptoms. Patients with chronic nasal disease or recent upper respiratory tract infection were not included in the final analytic cohort. TNM stage was classified according to the eighth edition of the AJCC TNM staging system. Surgical procedures were categorized as thyroidectomy alone, thyroidectomy with central neck dissection, and thyroidectomy with lateral neck dissection.

### Intervention and standard care

Standard care included hydration guidance, consumption of acid-containing food or drink to promote salivary secretion, oral hygiene measures, and radiation-safety counseling. All patients received the same standard RAI-related nursing education. Hydration guidance required fluid intake of more than 3,000 mL/day during the first 7 days after ^131^I administration and maintenance of more than 2,000 mL/day from day 8 until the 2-month post-discharge follow-up, unless medically contraindicated. Nasal irrigation was not part of standard care in the control group.

In addition to standard care, patients in the irrigation group followed a stepped nasal irrigation protocol. Nasal irrigation education was provided before ^131^I administration as part of the treatment-admission nursing education. Patients in the irrigation group additionally received oral and written instructions on nasal irrigation. Trained nurses demonstrated the procedure at bedside and asked patients to practice until they could perform the procedure independently and correctly. Throughout the inpatient stay and for the first month after discharge, 0.9% saline (120 mL per nostril) was delivered twice daily via a dedicated nasal irrigation device. Beginning in the second post-discharge month, the frequency was reduced to once daily until completion of the 2-month follow-up.

Adherence to nasal irrigation was assessed using routine nursing records and structured telephone follow-up. In the final analytic irrigation cohort, all 93 patients completed the prespecified irrigation protocol. Patients who discontinued or could not tolerate irrigation before completion of the prespecified protocol were not included in the efficacy cohort but were recorded in the screening and tolerability summary. Irrigation-related discomfort or adverse events, including nasal irritation, epistaxis, choking sensation, ear fullness or ear distension, poor tolerance, and discontinuation, were extracted from nursing and follow-up records when documented.

### Rescue medication protocol

A uniform rescue medication protocol applied to both groups. When any nasal symptom score reached ≥3 during follow-up, compound menthol nasal drops were prescribed at one to three applications daily. If this did not provide adequate relief, topical petrolatum ointment was added to support mucosal lubrication. Medication was withdrawn once symptoms resolved or on the basis of clinical and nursing judgment.

Because many patients did not require rescue medication, rescue medication use was analyzed primarily as the number and percentage of patients who received any rescue medication. Duration of medication use and total medication dose count were summarized as secondary medication-burden measures. Patients who did not receive rescue medication were assigned zero values for duration and total dose count, whereas time to first rescue medication was interpreted only among patients who actually received rescue medication.

### Clinical outcome assessment

Nasal symptom data were collected at four post-treatment time points: at discharge, and 2 weeks, 1 month, and 2 months after discharge. Pre-RAI nasal status was documented at baseline to exclude patients with pre-existing complaints. All follow-up assessments were conducted via structured telephone interviews, during which patients reported symptom scores verbally to trained nursing staff.

The symptom domains were nasal dryness (ND), nasal congestion (NC), nasal pain (NP), epistaxis (EP), and olfactory dysfunction (OD). Each symptom was scored using a 0–10 visual analog scale (VAS): 0 indicated no discomfort, 1–3 mild discomfort, 4–6 moderate discomfort, and 7–10 severe discomfort. The prespecified binary symptom endpoint was defined as any nasal symptom score ≥3 at any follow-up time point. Because a VAS score of 3 lies within the mild symptom range, this endpoint was described as a prespecified symptomatic-event threshold rather than definitive moderate-to-severe disease. A sensitivity analysis using VAS ≥4 was planned to evaluate moderate-or-worse nasal symptoms. The total symptom score was calculated as the sum of the five domain scores.

### Imaging assessment and T/B ratio calculation

Post-therapy whole-body imaging was performed on days 3 and 7 after ^131^I administration using a PHILIPS Precedence SPECT/CT system. The acquisition range extended from the skull to the feet, with a 256 × 1024 matrix and a scanning speed of 15 cm/min. These two imaging time points were treated as early- and delayed-phase assessments for quantitative T/B analysis. On both imaging days, routine post-therapy scintigraphy was completed before the scheduled nasal irrigation session on the same day. Therefore, the day-3 and day-7 T/B measurements were not obtained immediately after lavage.

For quantitative analysis, regions of interest (ROIs) were drawn on post-therapy planar whole-body scintigraphy images. The nasal cavity and nasopharyngeal region were defined as the target region. Circular or freehand ROIs were used, with placement chosen to cover the area of greatest radioiodine concentration within the nasal cavity/nasopharyngeal target region. A same-sized background ROI was placed over a non-avid craniofacial background region on the same image plane. The nasal mucosa target-to-background ratio (T/B ratio) was calculated as mean target counts divided by mean background counts.

ROI placement was performed independently by two nuclear medicine physicians who were blinded to group assignment and clinical symptom data. Disagreements in ROI placement were resolved by consensus, and the consensus T/B values were used for statistical analysis. A de-identified representative post-therapy image is included as **Figures 1C, D** to illustrate target and background ROI placement. The T/B ratio was considered an exploratory imaging surrogate of local nasal radioiodine retention rather than a direct dosimetric measure of absorbed radiation dose.

**Figure 1:**
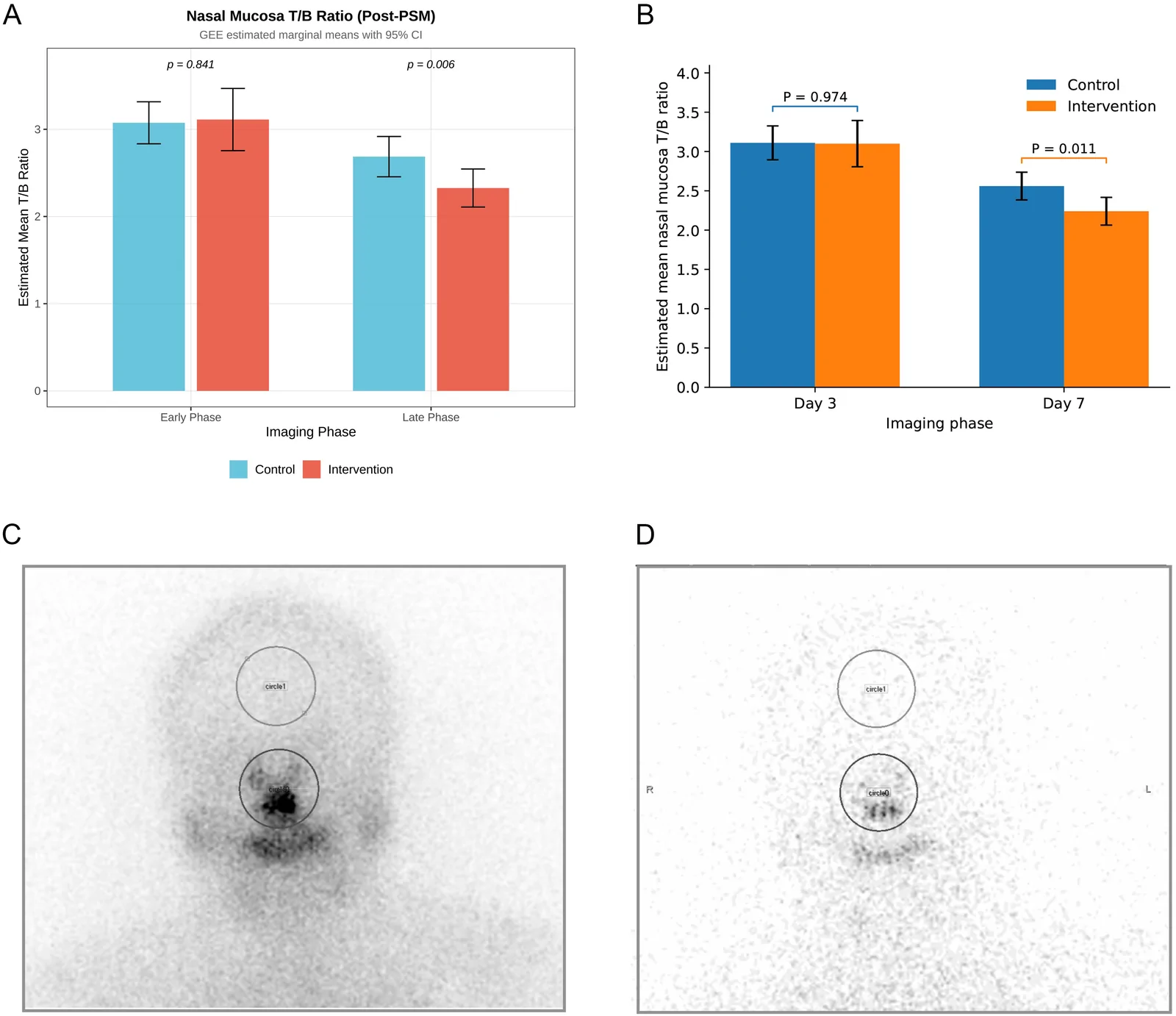
Imaging-based assessment of nasal radioiodine retention. **(A)** Generalized estimating equation-derived estimated marginal means of nasal mucosa target-to-background (T/B) ratios in the early and late imaging phases after propensity score matching. **(B)** Mean nasal mucosa T/B ratios on days 3 and 7 after ^131^I administration in the matched cohort. Error bars indicate 95% confidence intervals. **(C, D)** Representative de-identified post-therapy whole-body images showing ROI placement in the nasal cavity/nasopharyngeal target region and adjacent background region. Panel **(C)** shows the day-3 image, and panel **(D)** shows the day-7 image.

### Analysis sets, missing data, and model inclusion

The original retrospective cohort comprised all eligible patients included after screening. The matched cohort was constructed using propensity score matching and served as the primary analysis cohort. For each endpoint, patients were included in the corresponding statistical model if the required outcome and covariate data were available. The supplementary analysis-set table summarizes patient screening, exclusions, cohort construction, imaging and rescue-medication availability, and model-specific sample sizes.

Variables relevant to potential selection bias or confounding, including ^131^I activity, number of prior ^131^I treatments, smoking history, length of inpatient stay, hydration eligibility, TSH stimulation method, pre-existing nasal disease, recent upper respiratory tract infection, and baseline nasal symptoms, were extracted when available and reported or considered in sensitivity analyses as appropriate. Variables that were unavailable in the retrospective database were not imputed.

To evaluate potential bias arising from the exclusion of patients who discontinued or could not tolerate nasal irrigation, we performed a conservative as-initially-managed sensitivity analysis. This analysis included all 101 patients initially managed with nasal irrigation guidance, and the eight non-completers were counted as events for the binary symptom and rescue-medication endpoints. Because treatment assignment was not randomized, this analysis should be regarded as a pragmatic, treatment-strategy-based approach rather than a formal intention-to-treat analysis in the strict statistical sense.

### Propensity score matching and statistical analysis

All statistical analyses were performed using R software version 4.4.1. Continuous variables were summarized as mean ± standard deviation or median (interquartile range), according to distribution. Demographic and baseline continuous variables were rounded to one decimal place where appropriate, consistent with the precision of the source data. Categorical variables were summarized as counts and percentages. Between-group comparisons used the t test, Wilcoxon rank-sum test, chi-square test, or Fisher exact test, as appropriate.

Propensity scores were estimated using logistic regression with treatment group as the dependent variable. Matching was performed with the MatchIt package using 1:1 nearest-neighbor matching with a caliper of 0.1 and without replacement. In the revised propensity score model, covariates were selected on the basis of clinical relevance and plausible association with both treatment allocation and post-RAI nasal symptoms. The model included age, sex, TNM stage, surgical procedure, and administered ^131^I activity and number of ^131^I treatments, both of which were incorporated given their established biological relevance to radiation-related toxicity. Smoking history and length of inpatient stay were not included in the primary model because of incomplete data ascertainment; where available, these variables were evaluated in supplementary balance and sensitivity analyses. Hydration ineligibility, pre-existing nasal disease, recent upper respiratory tract infection, and baseline nasal symptoms were addressed through eligibility criteria rather than statistical adjustment. Because all patients underwent endogenous TSH stimulation without recombinant human TSH (rhTSH), TSH stimulation strategy did not vary across the cohort and was therefore not entered as a covariate in the model. Additional clinically relevant variables, including smoking history and length of inpatient stay, were assessed in the revised analysis when available. Hydration ineligibility, pre-existing nasal disease, recent upper respiratory tract infection, and baseline nasal symptoms were handled at the eligibility-screening stage. Covariate balance was assessed using standardized mean differences (SMDs), with SMD <0.1 considered good balance and SMD <0.2 considered acceptable.

For binary clinical outcomes, generalized linear models with a logit link and binomial distribution were used, and adjusted risk differences (RDs) with 95% confidence intervals (CIs) were estimated using the marginaleffects package. Adjusted models included age, sex, TNM stage, surgical procedure, ^131^I activity, and number of ^131^I treatments. The primary binary analysis used the prespecified VAS ≥3 threshold, and a sensitivity analysis using VAS ≥4 was performed to assess moderate-or-worse symptoms. For longitudinal symptom scores and T/B ratios, generalized estimating equations (GEE) with an exchangeable correlation structure were used. Models included group, time, and the group-by-time interaction, with adjustment for the same clinical covariates when model convergence allowed. Estimated marginal means were extracted using the emmeans package. Spearman rank correlation was used to assess associations between nasal mucosa T/B ratios and symptom scores. Two-sided P<0.05 was considered statistically significant.

## Results

### Patient flow, analysis sets, and baseline characteristics

A total of 208 patients were screened during the institutional project period. Twenty-three patients were excluded before construction of the final retrospective cohort: four because adequate hydration could not be ensured owing to cardiopulmonary conditions, eight because of pre-existing nasal disease, two because of nasal symptoms during the treatment admission, eight because of irrigation-related discontinuation or inability to complete the prespecified irrigation protocol, and one because of loss to follow-up. The final retrospective cohort therefore included 185 eligible patients, with 92 receiving standard care and 93 receiving standard care plus stepped nasal irrigation (**Figures 1A**, **2A**).

**Figure 2:**
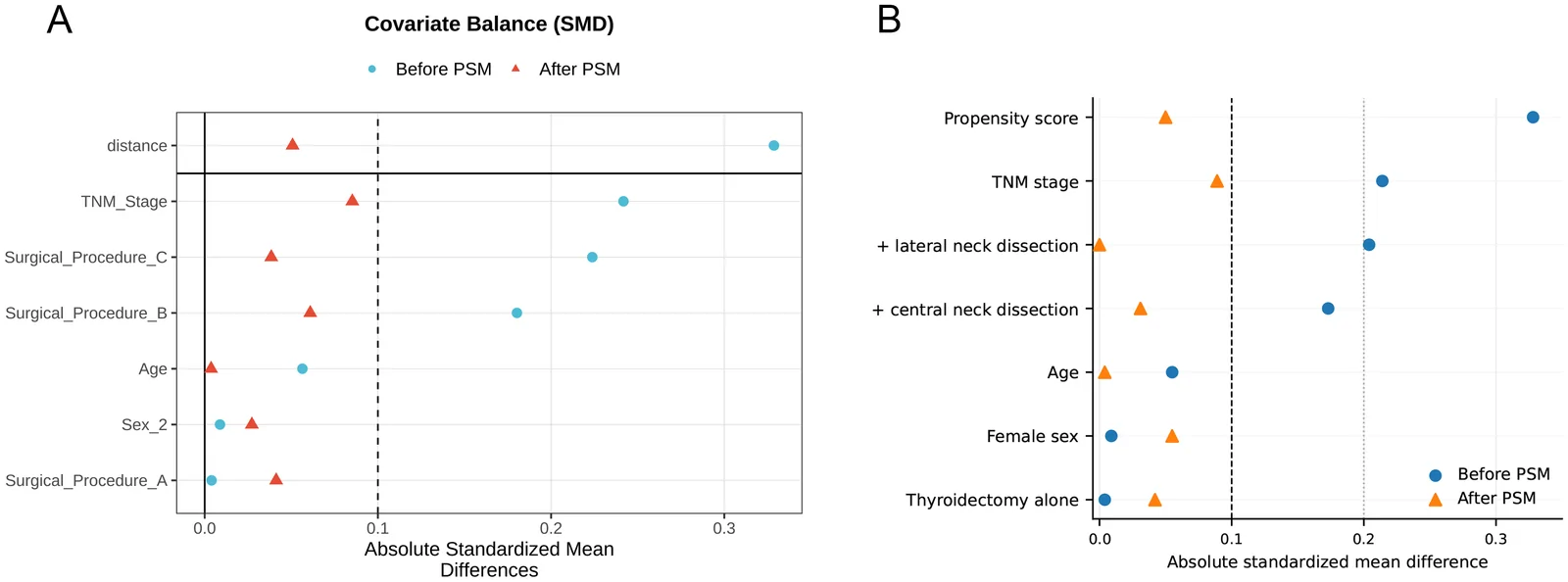
Study profile and covariate balance. **(A)** Flow diagram of patient screening, group assignment, propensity score matching, and analysis sets. **(B)** Absolute standardized mean differences for baseline covariates before and after propensity score matching. The dashed vertical line indicates an SMD of 0.10.

The mean age of the full cohort was 46.2 ± 14.6 years. **Table 1** presents the pre-matching baseline characteristics, including age, sex, pathological subtype, TNM stage, surgical extent, administered ^131^I activity, and number of prior ^131^I treatments. Additional variables relevant to potential confounding and selection bias are summarized in **Supplementary Table 2**, including smoking history, length of inpatient stay, hydration eligibility, TSH stimulation method, pre-existing nasal disease, recent upper respiratory tract infection, baseline nasal symptoms, imaging availability, rescue-medication data, and model-specific sample sizes. In the original cohort, smoking history was documented in 8 of 92 patients (8.7%) in the standard-care group and 13 of 93 patients (14.0%) in the nasal irrigation group; the median length of inpatient stay was 9 days in both groups.

**Table 1:** Baseline characteristics of the original retrospective cohort.

Variables	Total (N = 185)	Control (N = 92)	Intervention (N = 93)	Statistic	P value
Age, mean ± SD	46.2 ± 14.6	46.6 ± 14.9	45.8 ± 14.4	t = 0.378	0.706
Sex, n (%)				χ² = 0.004	0.952
Male	74 (40.0)	37 (40.2)	37 (39.8)		
Female	111 (60.0)	55 (59.8)	56 (60.2)		
TNM stage, n (%)				χ² = 2.267	0.519
Stage 1	128 (69.2)	60 (65.2)	68 (73.1)		
Stage 2	38 (20.5)	20 (21.7)	18 (19.4)		
Stage 3	4 (2.2)	2 (2.2)	2 (2.2)		
Stage 4	15 (8.1)	10 (10.9)	5 (5.4)		
Tumor type, n (%)				χ² = -	–
Papillary thyroid carcinoma	185 (100.0)	92 (100.0)	93 (100.0)		
Surgical procedure, n (%)				χ² = 1.972	0.373
Thyroidectomy alone	22 (11.9)	11 (12.0)	11 (11.8)		
+ central neck dissection	130 (70.3)	61 (66.3)	69 (74.2)		
+ lateral neck dissection	33 (17.8)	20 (21.7)	13 (14.0)		
^131^I activity (mCi), mean ± SD	158.89 ± 27.39	161.25 ± 29.12	156.56 ± 25.51	t = 1.166	0.245
Number of ^131^I treatments, mean ± SD	1.50 ± 1.18	1.51 ± 1.18	1.48 ± 1.19	t = 0.155	0.877

^131^I, iodine-131; SD, standard deviation; PSM, propensity score matching. The table describes the original cohort (N = 185; standard care n=92; nasal irrigation n=93). Matched-cohort balance is shown in **Figure 2B**.

The distribution of propensity scores showed improved overlap between groups after matching, supporting acceptable common support for the revised matched analysis (**Supplementary Figure 1**).

After PSM, the matched cohort included 148 patients, with 74 patients in each group. In the matched cohort, the median age was 42.0 years (IQR, 35.0–54.3), and 88 patients (59.5%) were female. Baseline variables were well balanced after matching, including age (P = 0.655), sex (P = 0.738), TNM stage (P = 0.947), surgical procedure (P = 0.967), ^131^I activity (P = 0.670), and number of ^131^I treatments (P = 0.809; **Table 2** and **Figure 2B**). All 148 matched patients contributed to the primary binary symptom analysis, longitudinal symptom-score analysis, rescue-medication burden analysis, and imaging-based T/B ratio analysis.

**Table 2:** Baseline characteristics of the propensity-score-matched cohort.

Analysis	Cohort	Standard care	Nasal irrigation	Notes
Primary binary symptom analysis, VAS ≥3	Matched cohort	74	74	Main analysis
Longitudinal symptom-score GEE	Matched cohort	74	74	Four follow-up time points
Rescue-medication burden analysis	Matched cohort	74	74	Duration and total dose count
Imaging T/B ratio analysis	Matched cohort	74	74	Day 3 and day 7
Rescue medication use	Original cohort	30/92 (32.6%)	13/93 (14.0%)	n/%
Rescue medication use	Matched cohort	25/74 (33.8%)	10/74 (13.5%)	n/%
VAS ≥4 sensitivity analysis	Original cohort	29/92 (31.5%)	13/93 (14.0%)	Exploratory; not PSM-based

After expanded PSM incorporating age, sex, TNM stage, surgical procedure, administered ^131^I activity, and number of ^131^I treatments, the matched cohort included 148 patients, with 74 patients in each group. Covariate balance was improved after matching, with a maximum absolute SMD of 0.114. The distribution of propensity scores showed improved overlap between groups after matching, supporting acceptable common support for the revised matched analysis (**Supplementary Figure 1**).

The distribution of propensity scores before and after matching was plotted to visually assess common support and improvement in between-group overlap.

### Adherence and tolerability of nasal irrigation

Among 101 patients initially managed with nasal irrigation guidance, 93 patients (92.1%) completed the prespecified irrigation protocol and entered the final irrigation cohort. Eight patients (7.9%) discontinued irrigation or were unable to complete the prespecified protocol and were excluded before the final efficacy analysis. Documented reasons for discontinuation were persistent nasal mucosal irritation during irrigation in two patients, recurrent ear fullness or ear distension after irrigation in two patients, subjective lack of perceived benefit in one patient, and self-discontinuation after complete symptom relief in three patients on days 11, 18, and 20 after ^131^I administration. No patient was recorded as completing the protocol with a reduced irrigation frequency. Symptoms related to irritation or ear discomfort gradually resolved after discontinuation or correction of the irrigation technique. No severe irrigation-related adverse event was documented in the available nursing or follow-up records.

### Incidence of nasal symptoms after PSM

Nasal irrigation was associated with a lower incidence of nasal symptoms meeting the prespecified VAS ≥3 threshold in the matched cohort. Symptoms meeting this threshold occurred in 27 of 74 patients (36.5%) receiving standard care and in 13 of 74 patients (17.6%) undergoing nasal irrigation (RD, −20.27 percentage points; 95% CI, −34.32 to −6.22; P = 0.005). Individual symptom-domain incidence rates are shown in **Figure 3** and **Supplementary Table 1**.

**Figure 3:**
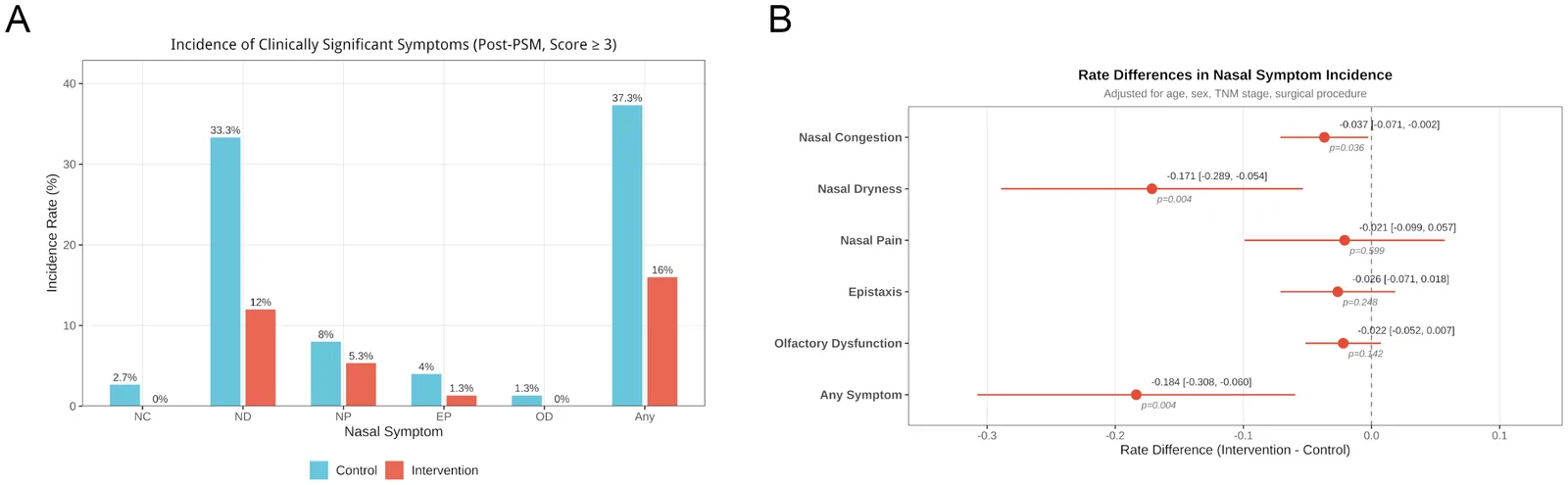
Incidence of nasal symptoms meeting the prespecified VAS ≥3 threshold after propensity score matching. **(A)** Incidence rates of nasal congestion (NC), nasal dryness (ND), nasal pain (NP), epistaxis (EP), olfactory dysfunction (OD), and any nasal symptom after propensity score matching. **(B)** Risk differences with 95% confidence intervals comparing nasal irrigation with standard care in the matched cohort.

Because a VAS score of 3 represents the upper range of mild discomfort, we performed an exploratory sensitivity analysis in the original retrospective cohort using VAS ≥4 to define moderate-or-worse nasal symptoms. Moderate-or-worse nasal symptoms occurred in 29 of 92 patients (31.5%) in the standard-care group and 13 of 93 patients (14.0%) in the nasal irrigation group, showing the same direction of association as the primary matched analysis (unadjusted RD, −17.54 percentage points; 95% CI, −29.37 to −5.72; Fisher exact P = 0.005).

### Rescue medication use

Rescue medication use was first analyzed as the number and percentage of patients who required medication. In the original retrospective cohort, rescue medication was used by 30 of 92 patients (32.6%) in the standard-care group and 13 of 93 patients (14.0%) in the nasal irrigation group (unadjusted RD, −18.63 percentage points; 95% CI, −30.52 to −6.74; Fisher exact P = 0.003). After PSM, rescue medication was required in 25 of 74 patients (33.8%) in the standard-care group and 10 of 74 patients (13.5%) in the nasal irrigation group (unadjusted RD, −20.27 percentage points; 95% CI, −33.57 to −6.97; Fisher exact P = 0.006).

The overall distribution of rescue-medication burden was lower in the nasal irrigation group after PSM. The median duration of rescue medication use was 0.00 days (IQR, 0.00–4.50) in the standard-care group and 0.00 days (IQR, 0.00–0.00) in the nasal irrigation group (z=2.961, P = 0.003). The median total dose count was also lower in the irrigation group: 0.00 (IQR, 0.00–0.00) versus 0.00 (IQR, 0.00–18.00) in the standard-care group (z=3.022, P = 0.003; **Figure 4**). Because patients who did not receive rescue medication had zero values for medication-related variables, these results primarily reflect a lower overall need for rescue treatment rather than a delayed time to medication among all patients. The recorded time to first rescue medication is therefore shown descriptively in **Figure 4A** and was not interpreted as a true time-to-event endpoint for the whole cohort.

**Figure 4:**
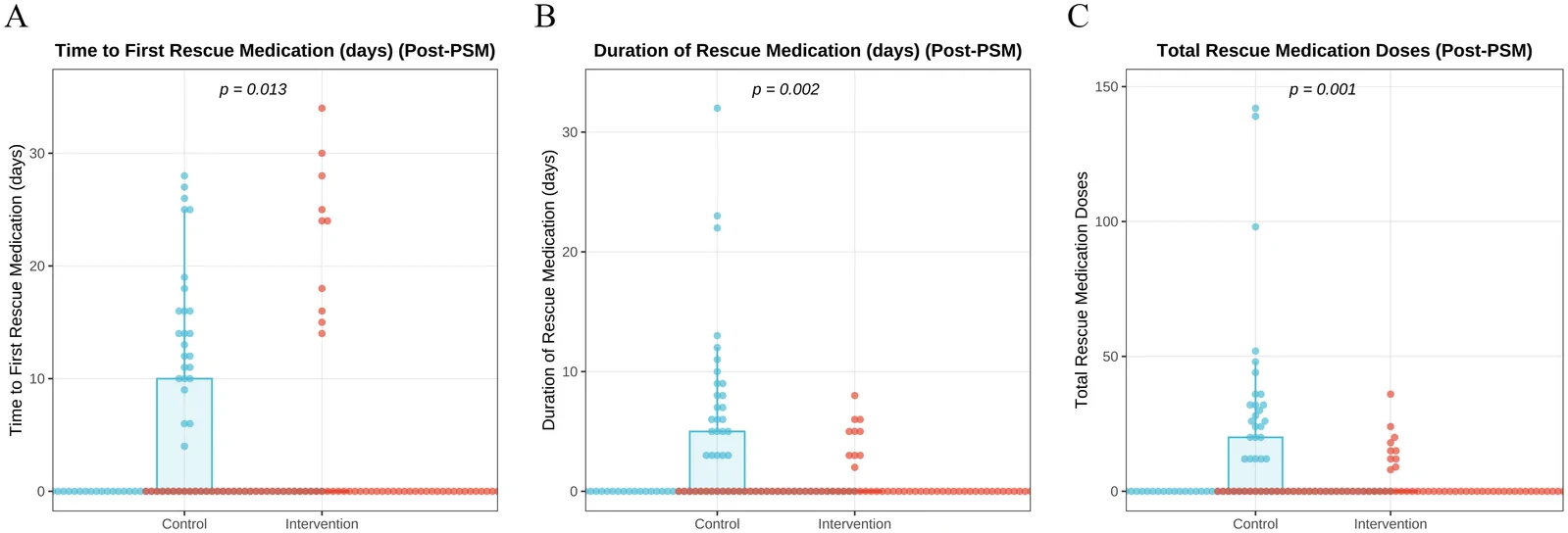
Rescue medication use after propensity score matching. **(A)** Time to first rescue medication. **(B)** Duration of rescue medication use. **(C)** Total rescue medication doses. P values were calculated using Wilcoxon rank-sum tests unless otherwise specified.

### Longitudinal symptom trajectories

Total symptom scores were consistently lower in the nasal irrigation group across all follow-up visits after PSM. Mean scores in the standard-care versus irrigation groups were 0.39 ± 0.99 versus 0.00 ± 0.00 at discharge (P = 0.001), 1.74 ± 2.75 versus 0.55 ± 1.42 at 2 weeks (P = 0.001), 1.74 ± 2.67 versus 0.69 ± 1.70 at 1 month (P = 0.005), and 0.64 ± 1.20 versus 0.19 ± 0.59 at 2 months (P = 0.005) (**Figure 5A**).

**Figure 5:**
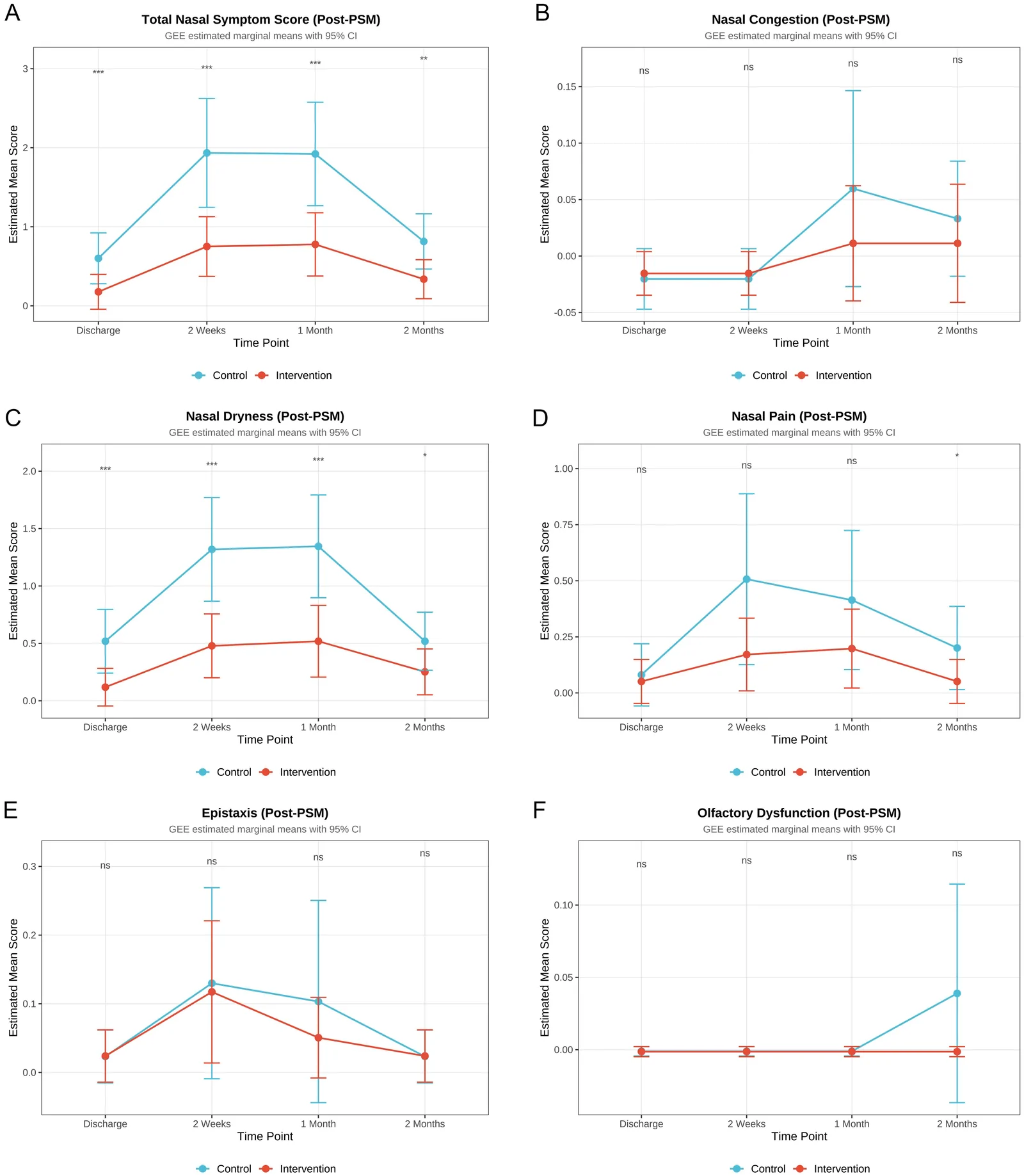
Longitudinal nasal symptom scores after propensity score matching. **(A)** Total nasal symptom score. **(B)** Nasal congestion (NC). **(C)** Nasal dryness (ND). **(D)** Nasal pain (NP). **(E)** Epistaxis (EP). **(F)** Olfactory dysfunction (OD). Estimated marginal means and 95% confidence intervals were derived from generalized estimating equation models across discharge, 2 weeks, 1 month, and 2 months after treatment.

Among individual domains, the most consistent difference was observed for nasal dryness. Mean nasal dryness scores were lower in the nasal irrigation group at discharge, 2 weeks, 1 month, and 2 months, and the GEE model for nasal dryness showed significant group (chi-square=14.092, P<0.001), time (chi-square=43.811, P<0.001), and group-by-time interaction effects (chi-square=12.579, P = 0.006). Differences in nasal pain were smaller and less consistent, whereas nasal congestion, epistaxis, and olfactory dysfunction had low absolute scores and limited between group separation (**Figures 5B–F**). These domain-level findings indicate that the observed clinical association was driven mainly by nasal dryness rather than by uniform improvement across all nasal symptom domains.

### Imaging-based assessment of nasal radioiodine retention

Post-therapy imaging on days 3 and 7 was used to assess nasal cavity/nasopharyngeal radioiodine retention. All day-3 and day-7 images included in the T/B analysis were obtained before the scheduled nasal irrigation session on the same imaging day. After PSM, the early-phase T/B ratio on day 3 was similar between the standard-care and nasal irrigation groups (3.11 ± 0.96 vs. 3.10 ± 1.34; P = 0.977). By day 7, the delayed-phase T/B ratio was lower in the nasal irrigation group (2.24 ± 0.76) than in the standard-care group (2.56 ± 0.81; P = 0.012) (**Figure 1A**).

GEE analysis confirmed a significant time effect (chi-square=39.714, P<0.001), while the overall group effect (chi-square=1.910, P = 0.167) and group-by-time interaction (chi-square=2.065, P = 0.151) did not reach statistical significance. In time-specific contrasts, the between-group difference was negligible on day 3 (difference, −0.01; 95% CI, −0.38 to 0.37; P = 0.974) but significant on day 7 (difference, −0.33; 95% CI, −0.58 to −0.08; P = 0.011; **Figure 1B**). Representative de-identified post-therapy whole-body images showing ROI placement are shown in **Figures 1C, D**. These imaging findings were treated as exploratory and were not interpreted as direct proof of enhanced radioiodine clearance.

Because neither the overall group effect nor the group-by-time interaction reached statistical significance in the longitudinal GEE model, the significant day-7 contrast was interpreted as exploratory rather than definitive evidence of a treatment-related reduction in radioiodine retention.

Symptoms meeting the prespecified VAS ≥3 threshold occurred in 27 of 74 patients (36.5%) receiving standard care and 13 of 74 patients (17.6%) undergoing nasal irrigation in the expanded matched cohort (RD, −18.92 percentage points; 95% CI, −32.90 to −4.94; P = 0.016). In the same expanded matched cohort, moderate-or-worse symptoms, defined as VAS ≥4, occurred in 24 of 74 patients (32.4%) and 11 of 74 patients (14.9%), respectively (RD, −17.57 percentage points; 95% CI, −30.96 to −4.17; P = 0.019). Rescue medication was required in 25 of 74 patients (33.8%) and 11 of 74 patients (14.9%), respectively (RD, −18.92 percentage points; 95% CI, −32.40 to −5.43; P = 0.012).

In a conservative sensitivity analysis using the as-initially-managed analysis set—all 101 patients initially managed with a nasal irrigation strategy, regardless of protocol completion—the eight non-completers were assumed to have met each symptom or rescue-medication endpoint (i.e., counted as events), providing the most conservative estimate of bias from their exclusion. Under this assumption, the associations were attenuated but remained directionally consistent: symptoms meeting the prespecified VAS ≥3 threshold occurred in 34 of 92 patients (37.0%) in the standard-care group versus 24 of 101 patients (23.8%) in the irrigation group (RD, −13.19 percentage points; Fisher’s exact P = 0.059); moderate-or-worse symptoms (VAS ≥4) occurred in 29 of 92 patients (31.5%) versus 21 of 101 patients (20.8%) (Fisher’s exact P = 0.102); and rescue medication was required in 30 of 92 patients (32.6%) versus 21 of 101 patients (20.8%) (Fisher’s exact P = 0.073). None of these conservative comparisons reached conventional statistical significance.

## Discussion

In this retrospective propensity-score-matched cohort of patients with differentiated thyroid cancer receiving ^131^I therapy, a stepped program of isotonic saline nasal irrigation was associated with fewer post-RAI nasal symptoms, lower rescue-medication use, and a lower delayed-phase nasal mucosa target-to-background (T/B) ratio. The absolute reduction in symptoms meeting the prespecified VAS ≥3 threshold was approximately 20 percentage points after matching. The symptom pattern was not uniform across all nasal domains; the clearest and most reproducible difference was observed for nasal dryness, whereas nasal congestion, epistaxis, and olfactory dysfunction occurred at low absolute levels. Given the retrospective design, these results should be interpreted as a clinical association rather than definitive evidence of prophylaxis.

Supportive-care needs in thyroid cancer differ substantially by histological subtype, reflecting differences in the underlying treatment pathway. RAI therapy remains an established component of postoperative risk-adapted management for selected patients with DTC, and treatment-related salivary, lacrimal, and nasal toxicity accordingly represent a recognized burden in this population. Medullary thyroid carcinoma, however, is managed along a fundamentally different pathway: surgery constitutes the primary treatment for localized disease, and systemic targeted therapies are reserved for advanced or progressive disease, with RAI playing no role in its management (29). The supportive-care considerations examined in this study therefore pertain specifically to RAI-treated DTC and should not be extrapolated to thyroid malignancies more broadly.

Nasal toxicity after RAI therapy remains less familiar than salivary and lacrimal toxicity, although several studies suggest that it is not rare. Although post-RAI nasal toxicity is less recognized than sialadenitis or xerophthalmia, accumulating evidence confirms it is far from rare. Prior cohorts have documented post-ablation nasal dryness, olfactory dysfunction, and dysgeusia occurring at rates rivaling lacrimal complications (12, 13, 15, 16), often co-occurring with ocular surface disease and xerostomia (14). Collectively, these data position nasal symptoms within a broader post-RAI craniofacial toxicity profile rather than as sporadic complaints (17). Rather than merely expanding this descriptive literature, our study advances the field by testing a pragmatic, supportive-care solution.

The biological rationale for nasal irrigation is plausible, but it remains incompletely proven. The sodium-iodide symporter is expressed in several extrathyroidal tissues, and radioiodine-related toxicity in salivary and lacrimal structures has been attributed to uptake and secretion of radioiodine outside thyroid tissue (6, 10, 11, 18, 19). Nasal or nasolacrimal localization of ^131^I on post-therapy imaging has also been reported, supporting the possibility that radioiodine-containing secretions may contact the nasal cavity or adjacent mucosal surfaces after treatment (17). In this setting, saline irrigation could reduce mucosal exposure by diluting and removing retained secretions, improving hydration of the epithelial surface, and supporting mucociliary clearance. This mechanism is consistent with the established use of saline irrigation in chronic rhinosinusitis, allergic rhinitis, and other inflammatory sinonasal conditions (22–27, 30–32). However, our study did not measure radioiodine activity in nasal irrigation effluent. Therefore, we cannot directly quantify the amount of radioiodine removed by irrigation, and the proposed clearance mechanism should be regarded as an explanatory hypothesis rather than a demonstrated pathway.

The imaging findings offer a hypothesis-generating rather than confirmatory signal. Although the day-7 T/B ratio was lower in the nasal irrigation group within the matched cohort—a difference not present at day 3—this time-specific contrast did not correspond to a significant overall group effect or group-by-time interaction in the longitudinal GEE model, and should therefore be interpreted as exploratory. One plausible explanation is that the effect of nasal irrigation on clearance of retained radioiodine-containing secretions is cumulative, such that a measurable difference becomes apparent only after repeated irrigations—by day 7 rather than the earlier day-3 assessment. This interpretation remains speculative, however, given that the T/B ratio is a semiquantitative imaging surrogate rather than a direct measure of absorbed dose or mucociliary clearance; as such, it cannot establish that nasal irrigation accelerated radioactive clearance.

These results are best considered hypothesis-generating and should be tested with prospective imaging protocols, dosimetry, or effluent radioactivity measurement.

The clinical effect was driven mainly by nasal dryness. This pattern is clinically coherent. Nasal dryness is likely to be influenced by surface dehydration, secretion retention, and epithelial irritation, all of which may respond to isotonic saline irrigation. Nasal pain showed a smaller and less stable difference, while congestion, epistaxis, and olfactory dysfunction were uncommon and had limited between-group separation. Therefore, the present data do not support a broad claim that nasal irrigation improves all nasal symptoms after RAI. A more precise interpretation is that nasal irrigation was associated with a reduction in the overall symptomatic-event burden, with the strongest signal in nasal dryness. The exploratory VAS ≥4 sensitivity analysis in the original cohort showed the same direction of association for moderate-or-worse symptoms, which partially addresses the concern that VAS ≥3 may capture mild discomfort. Because this sensitivity analysis was not based on the matched cohort, it should be viewed as supportive rather than confirmatory.

The rescue-medication findings should also be interpreted with care. After PSM, fewer patients in the irrigation group required rescue medication, and the overall distributions of medication duration and total dose count were lower. This supports the clinical relevance of the symptom findings, because rescue medication use is less dependent on small changes in symptom scores alone. However, the median values for medication-related variables were zero in both groups because many patients did not require any rescue medication. For this reason, the number and percentage of patients requiring medication are more informative than the median time to first medication in the whole cohort. Time to first rescue medication should not be interpreted as a conventional time-to-event endpoint when patients without medication are coded as zero.

The safety and tolerability profile was generally acceptable, although not perfect. Among patients initially managed with nasal irrigation guidance, most completed the prespecified protocol. A small number discontinued because of nasal mucosal irritation, ear fullness or distension, subjective lack of perceived benefit, or self-discontinuation after symptom relief. No severe irrigation-related adverse event was documented in available nursing or follow-up records. These findings are consistent with the low-risk nature of isotonic saline irrigation, but they also show that tolerability should not be assumed. Future studies should prospectively collect adverse events, reasons for non-adherence, irrigation technique errors, and patient burden.

Several limitations deserve emphasis. First, the study was retrospective and non-randomized. Group assignment reflected an institutional ward-based supportive-care practice rather than randomized allocation. Although patients were consecutively included and the original group sizes were similar because of the predefined approximately 1:1 care arrangement, selection bias remains possible. Propensity score matching improved balance for measured variables such as age, sex, TNM stage, surgical procedure, ^131^I activity, and number of ^131^I treatments, but it cannot address unmeasured confounding. Hydration behavior, home environment, humidity, self-initiated nasal care, occupational irritant exposure, and subtle baseline differences in nasal mucosal status may have influenced symptom reporting or response to irrigation.

Second, symptom outcomes were based on patient-reported VAS scores obtained by telephone. This approach reflects routine post-RAI follow-up and allowed repeated assessment over two months, but it is susceptible to recall bias and expectation bias, particularly because patients and nurses were not blinded to the supportive-care strategy. The original term “clinically significant symptom” has therefore been replaced with the more cautious phrase “symptoms meeting the prespecified VAS ≥3 threshold.” Although the VAS ≥4 sensitivity analysis was directionally consistent, prospective studies should include validated rhinological instruments, such as SNOT-22, together with structured olfactory testing and endoscopic evaluation.

Third, the imaging analysis was exploratory. ROI placement was performed by two blinded physicians using target and background regions on post-therapy imaging, and disagreements were resolved by consensus. This reduced subjective bias in the imaging endpoint, but the study did not retain separate quantitative measurements from each reader for formal interobserver-agreement analysis. In addition, the T/B ratio reflects relative local radioiodine retention rather than absorbed radiation dose. The absence of nasal irrigation effluent radioactivity measurements is an important mechanistic limitation, because such measurements would be needed to directly confirm physical removal of radioiodine-containing secretions.

Fourth, the study was conducted at a single center and used a specific stepped irrigation protocol. The findings may not apply to different RAI activities, different lengths of hospitalization, other hydration protocols, alternative irrigation devices, different saline volumes, or patients with pre-existing rhinitis or chronic rhinosinusitis. Follow-up was limited to two months, which captures the early symptom period but not late olfactory, lacrimal, or mucosal sequelae. The study also excluded patients who could not complete the irrigation protocol before the final efficacy analysis, which may have made the estimated effect more favorable than would be observed under a strict intention-to-treat design.

Despite these limitations, the results are clinically relevant. RAI-related nasal symptoms are usually not life-threatening, but they are uncomfortable, under-recorded, and may add to the burden of treatment recovery. The intervention studied here is inexpensive, easy to teach, and broadly familiar to nursing practice. The present data suggest that stepped isotonic saline nasal irrigation may reduce early post-RAI nasal symptom burden, particularly nasal dryness, and may reduce the need for rescue medication. These findings justify prospective confirmation rather than immediate causal claims. A multicenter randomized trial should stratify patients by administered and cumulative ^131^I activity, document baseline nasal status, record adherence and adverse events prospectively, and incorporate validated patient-reported outcomes, endoscopic assessment, mucociliary clearance testing, olfactometry, and imaging or dosimetric substudies. Until such evidence is available, nasal irrigation may be considered a promising supportive-care strategy, but its preventive effect should be regarded as preliminary.

## Data Availability

The original contributions presented in the study are included in the article/**Supplementary Material**. Further inquiries can be directed to the corresponding authors.

## Supplementary material

Supplementary Figure 1 Propensity score distributions before and after expanded propensity score matching. The estimated propensity score distributions for the standard-care and nasal-irrigation groups are shown before and after matching. The expanded matching model included age, sex, TNM stage, surgical procedure, administered ^131^I activity, and number of ^131^I treatments. Improved overlap after matching indicates better covariate comparability between the two groups.
