# Supplementary material for: Association of nasal irrigation with reduced nasal symptoms after radioiodine ablation in differentiated thyroid cancer: a retrospective cohort study with propensity score matching
Source: Front Endocrinol (Lausanne). 2026 Sep 24;17:1897010. doi: 10.3389/fendo.2026.1897010 (PMC13645713; doi:10.3389/fendo.2026.1897010)
Supplement: Supplementary file 2 [file DataSheet10.pdf]

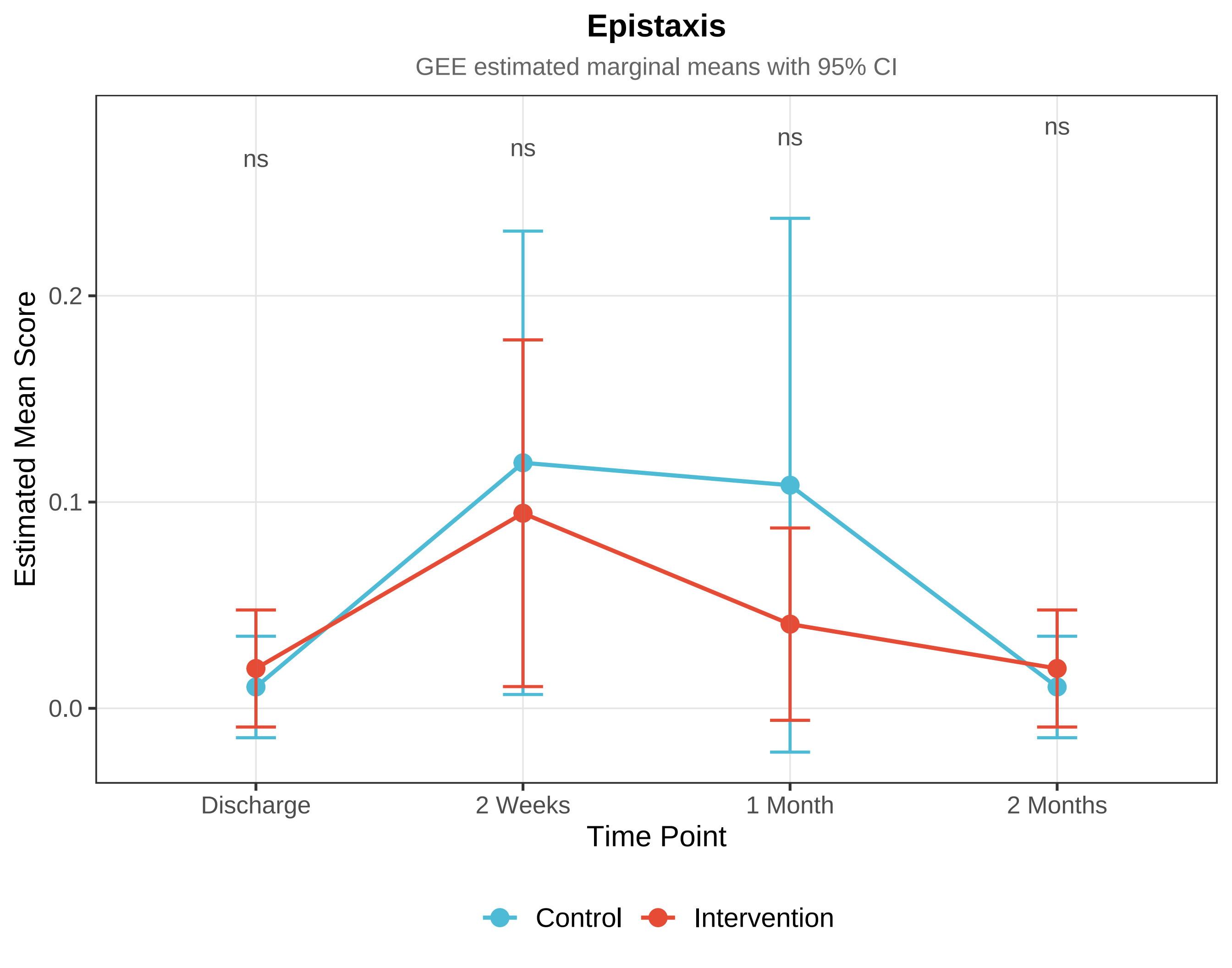
Epistaxis
GEE estimated marginal means with 95% CI
ns
ns
ns
ns
0.2
0.1
0.0
Estimated Mean Score
Discharge
2 Weeks
1 Month
2 Months
Time Point
Control
Intervention
