# Supplementary material for: Association of nasal irrigation with reduced nasal symptoms after radioiodine ablation in differentiated thyroid cancer: a retrospective cohort study with propensity score matching
Source: Front Endocrinol (Lausanne). 2026 Sep 24;17:1897010. doi: 10.3389/fendo.2026.1897010 (PMC13645713; doi:10.3389/fendo.2026.1897010)
Supplement: Supplementary file 4 [file DataSheet12.pdf]

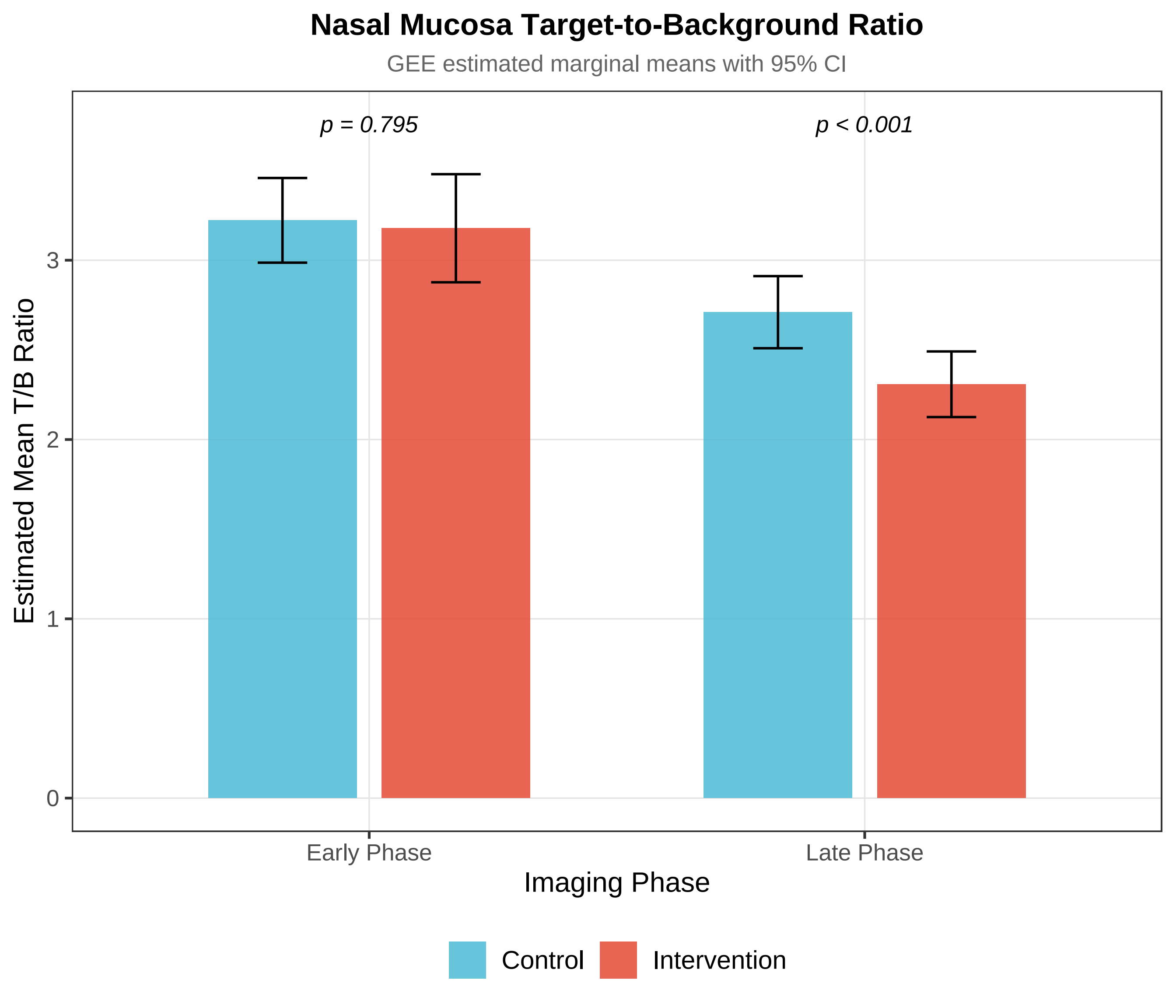
Nasal Mucosa Target-to-Background Ratio
GEE estimated marginal means with 95% CI
p = 0.795
p < 0.001
Estimated Mean T/B Ratio
0
1
2
3
Early Phase
Late Phase
Imaging Phase
Control
Intervention
