# Supplementary material for: Association of nasal irrigation with reduced nasal symptoms after radioiodine ablation in differentiated thyroid cancer: a retrospective cohort study with propensity score matching
Source: Front Endocrinol (Lausanne). 2026 Sep 24;17:1897010. doi: 10.3389/fendo.2026.1897010 (PMC13645713; doi:10.3389/fendo.2026.1897010)
Supplement: Supplementary file 5 [file DataSheet13.pdf]

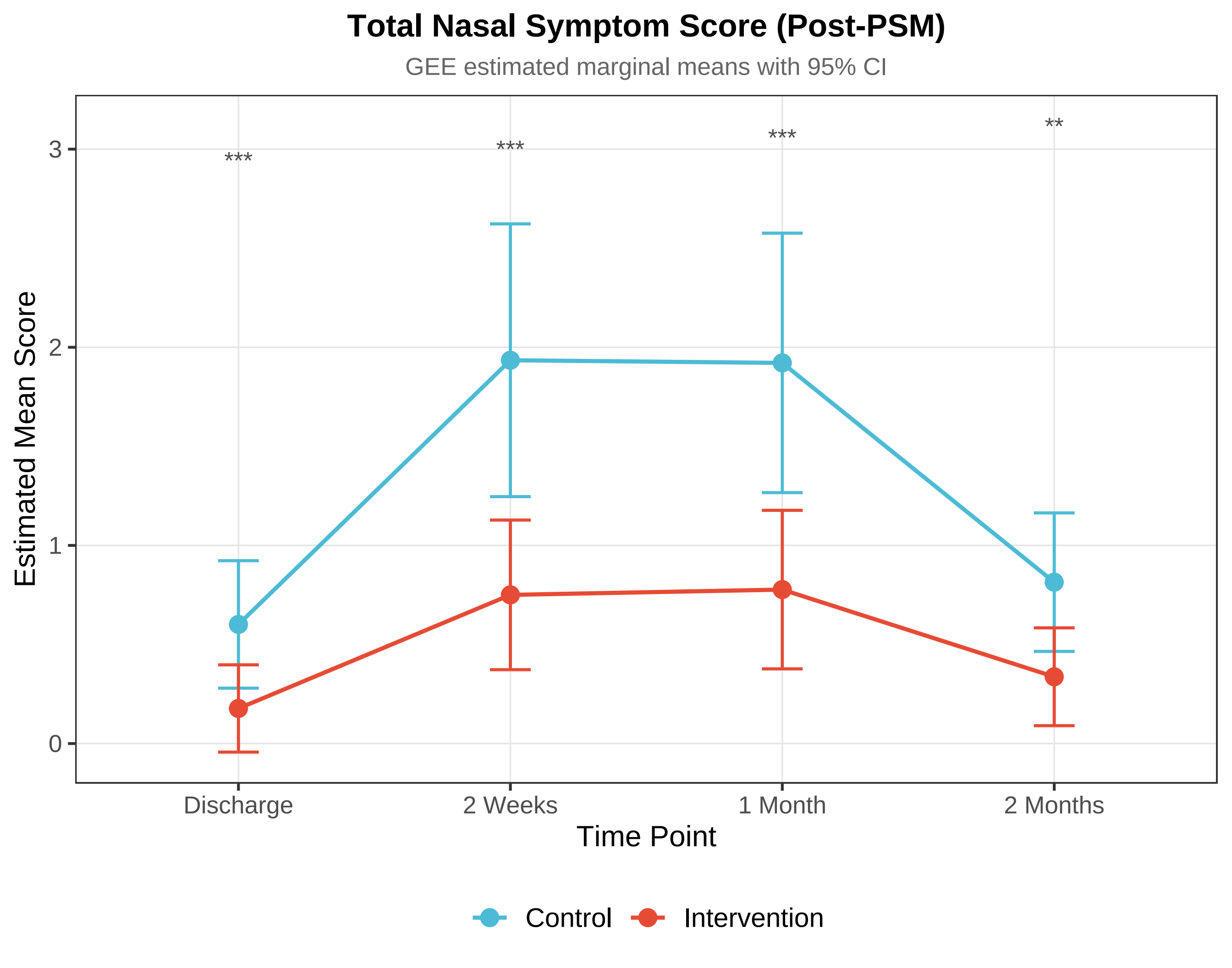

Total Nasal Symptom Score (Post-PSM)
GEE estimated marginal means with 95% CI
***
***
***
**
Estimated Mean Score
0
1
2
3
Discharge
2 Weeks
1 Month
2 Months
Time Point
Control
Intervention

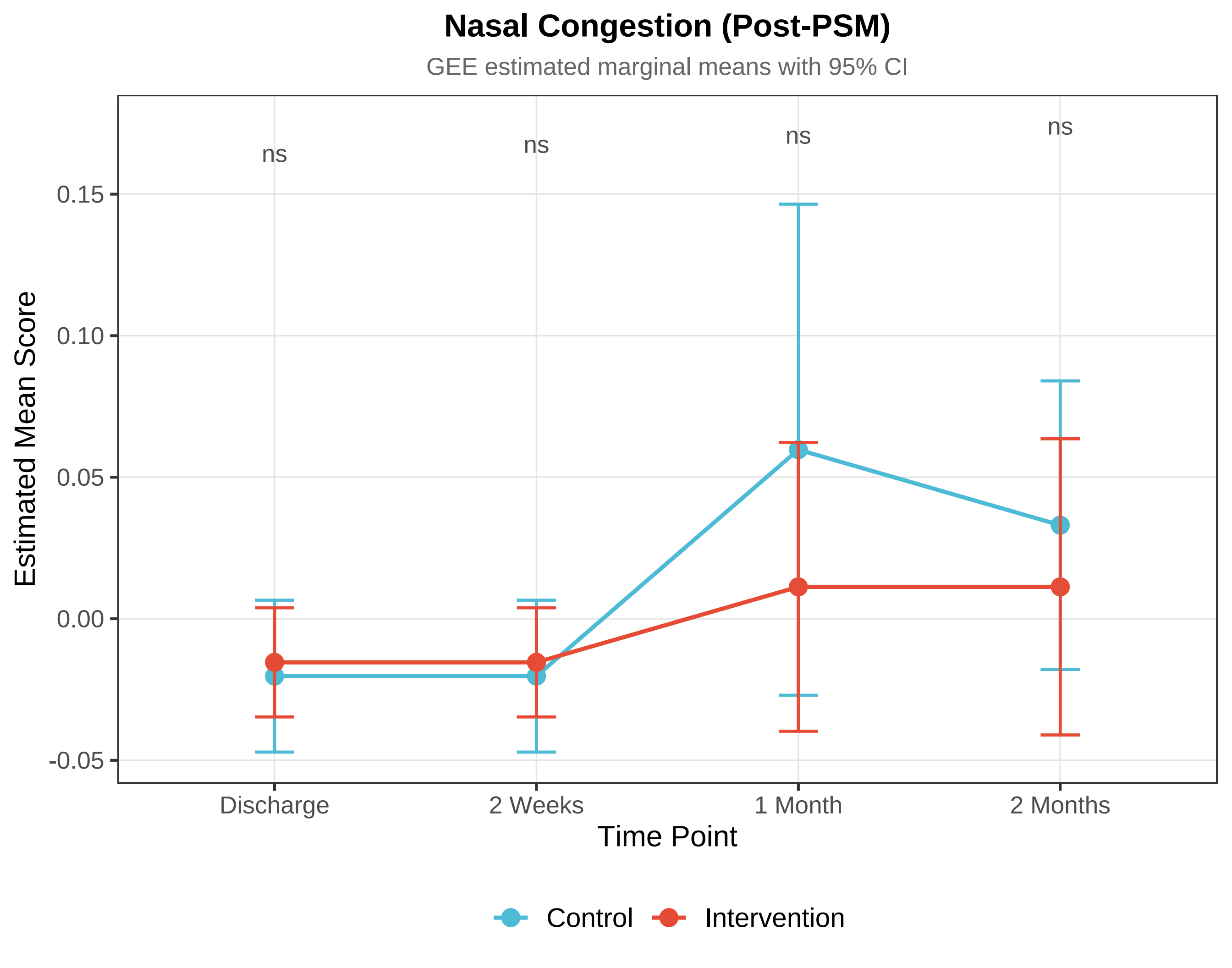
Nasal Congestion (Post-PSM)
GEE estimated marginal means with 95% CI
ns
ns
ns
ns
0.15
0.10
0.05
0.00
-0.05
Estimated Mean Score
Discharge
2 Weeks
1 Month
2 Months
Time Point
Control
Intervention

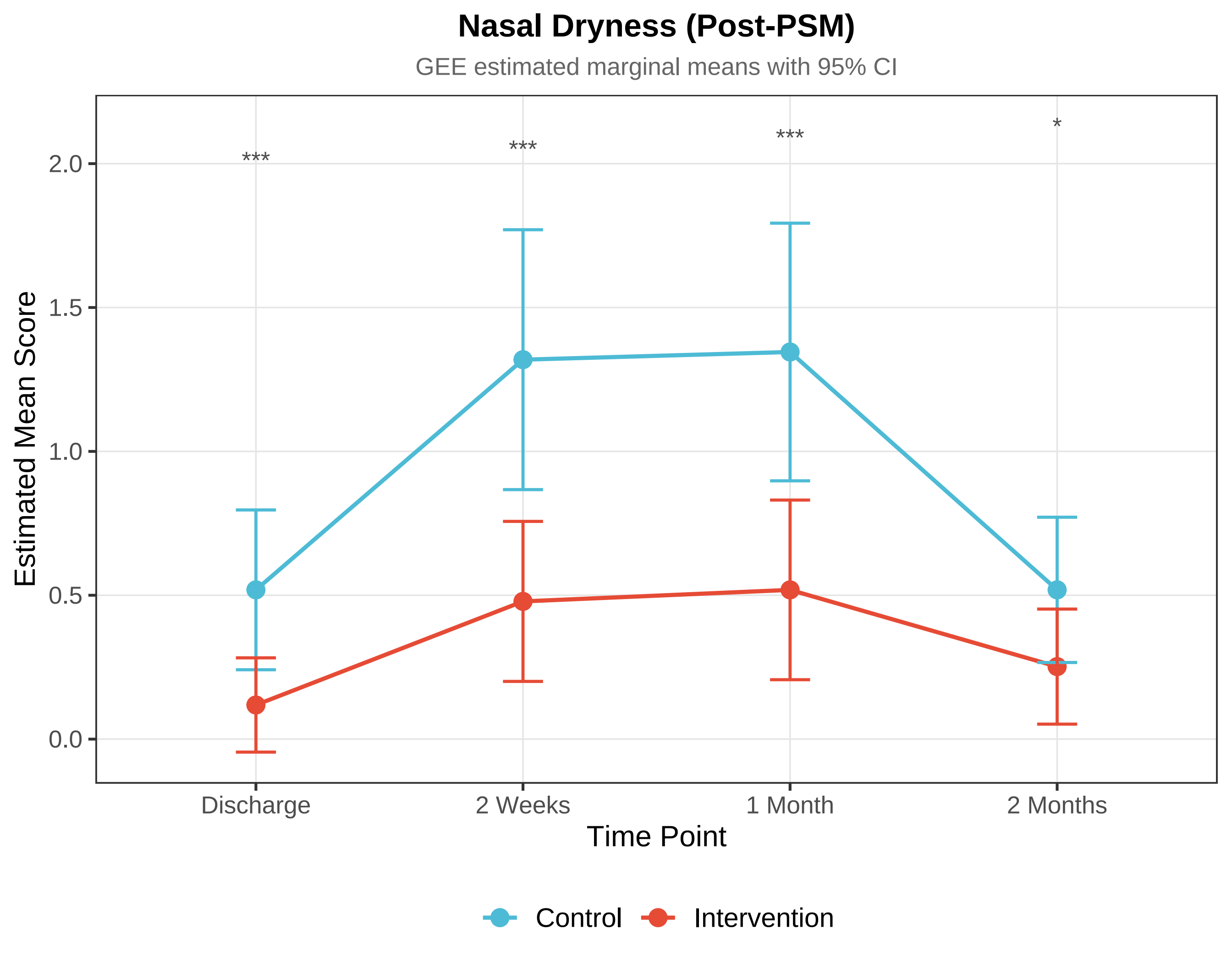

Nasal Dryness (Post-PSM)
GEE estimated marginal means with 95% CI
Estimated Mean Score
0.0
0.5
1.0
1.5
2.0
***
***
***
*
Discharge
2 Weeks
1 Month
2 Months
Time Point
Control
Intervention

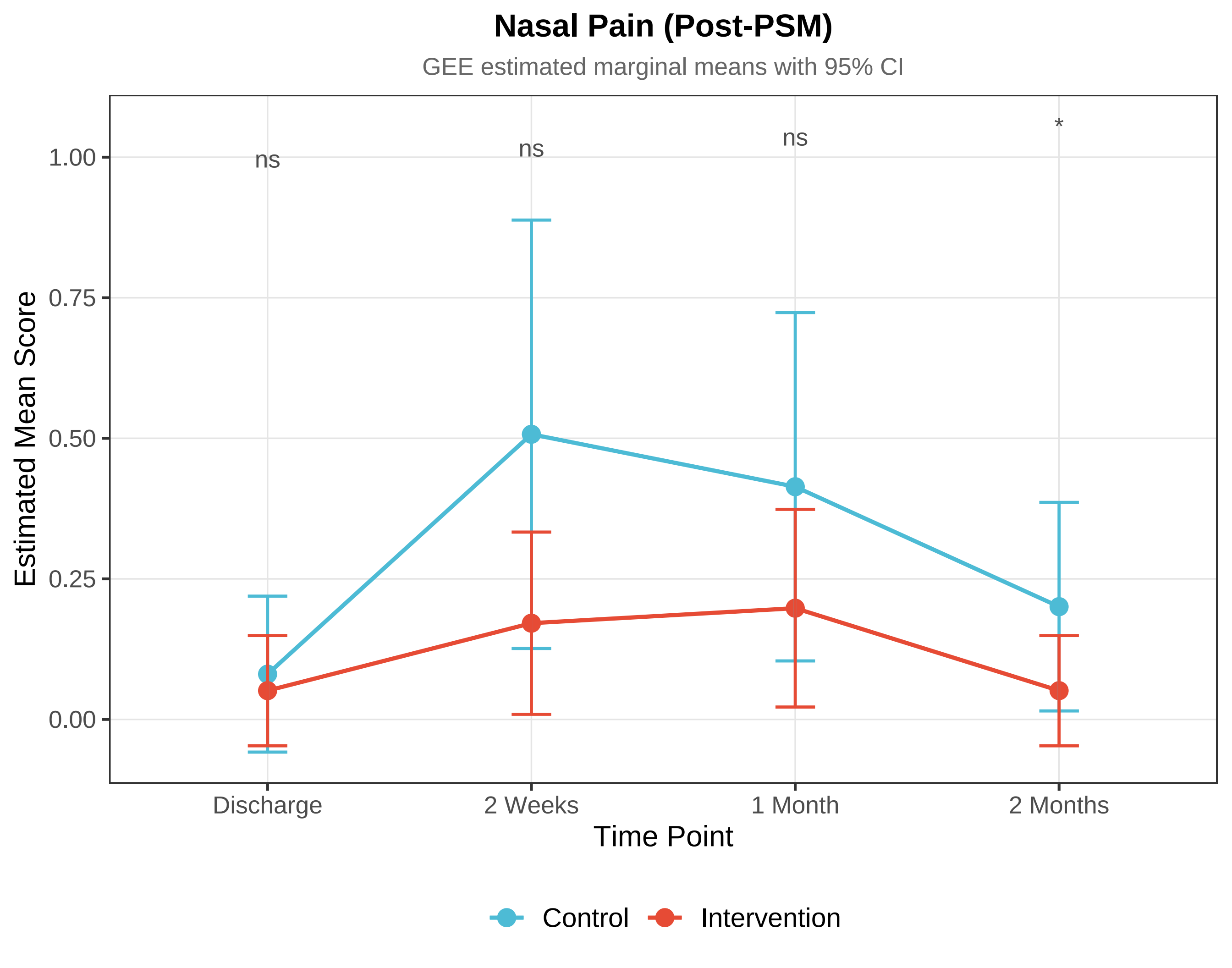

Nasal Pain (Post-PSM)
GEE estimated marginal means with 95% CI
ns
ns
ns
*
1.00
0.75
0.50
0.25
0.00
Estimated Mean Score
Discharge
2 Weeks
1 Month
2 Months
Time Point
Control
Intervention

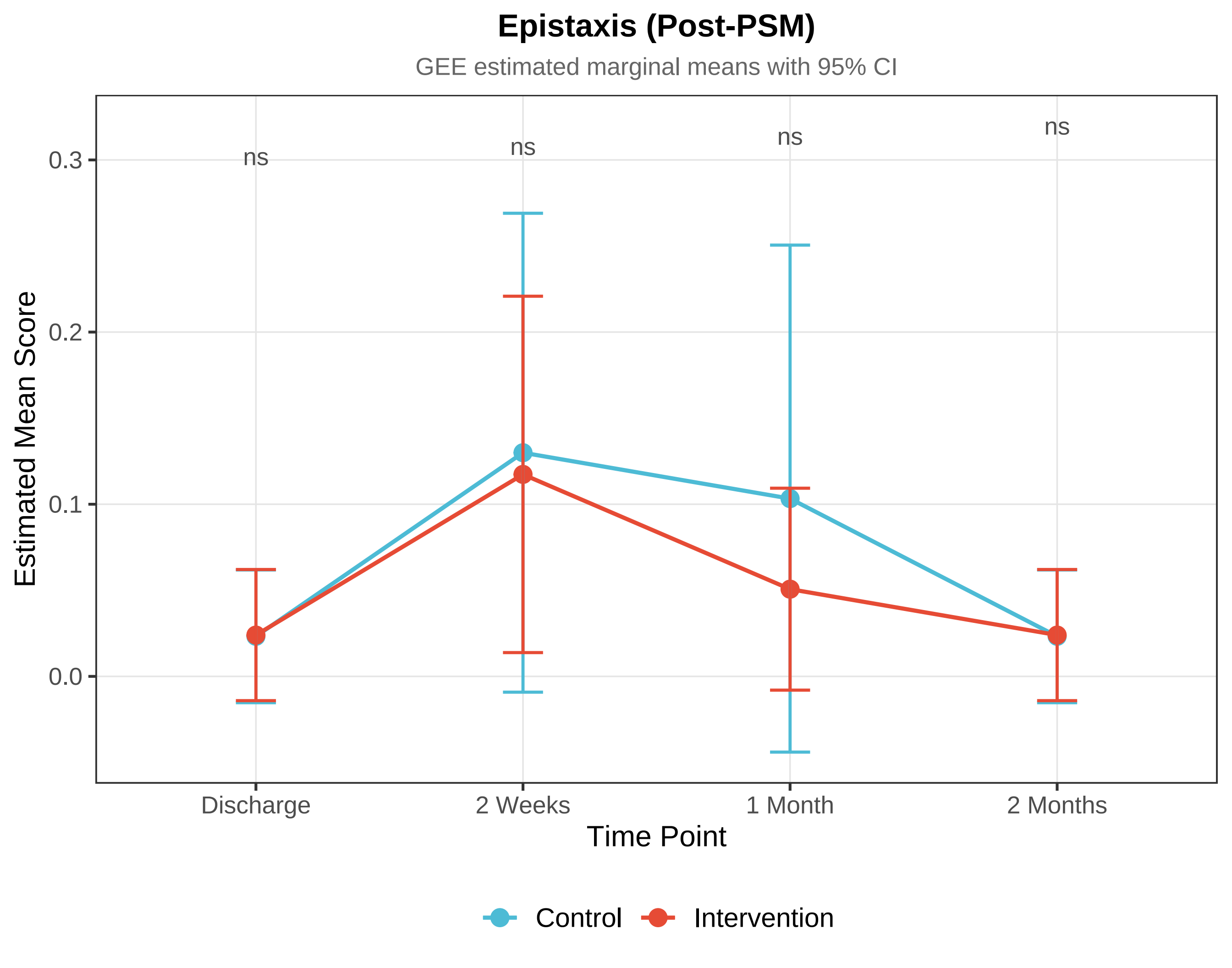
Epistaxis (Post-PSM)
GEE estimated marginal means with 95% CI
ns
ns
ns
ns
0.3
0.2
0.1
0.0
Estimated Mean Score
Discharge
2 Weeks
1 Month
2 Months
Time Point
Control
Intervention

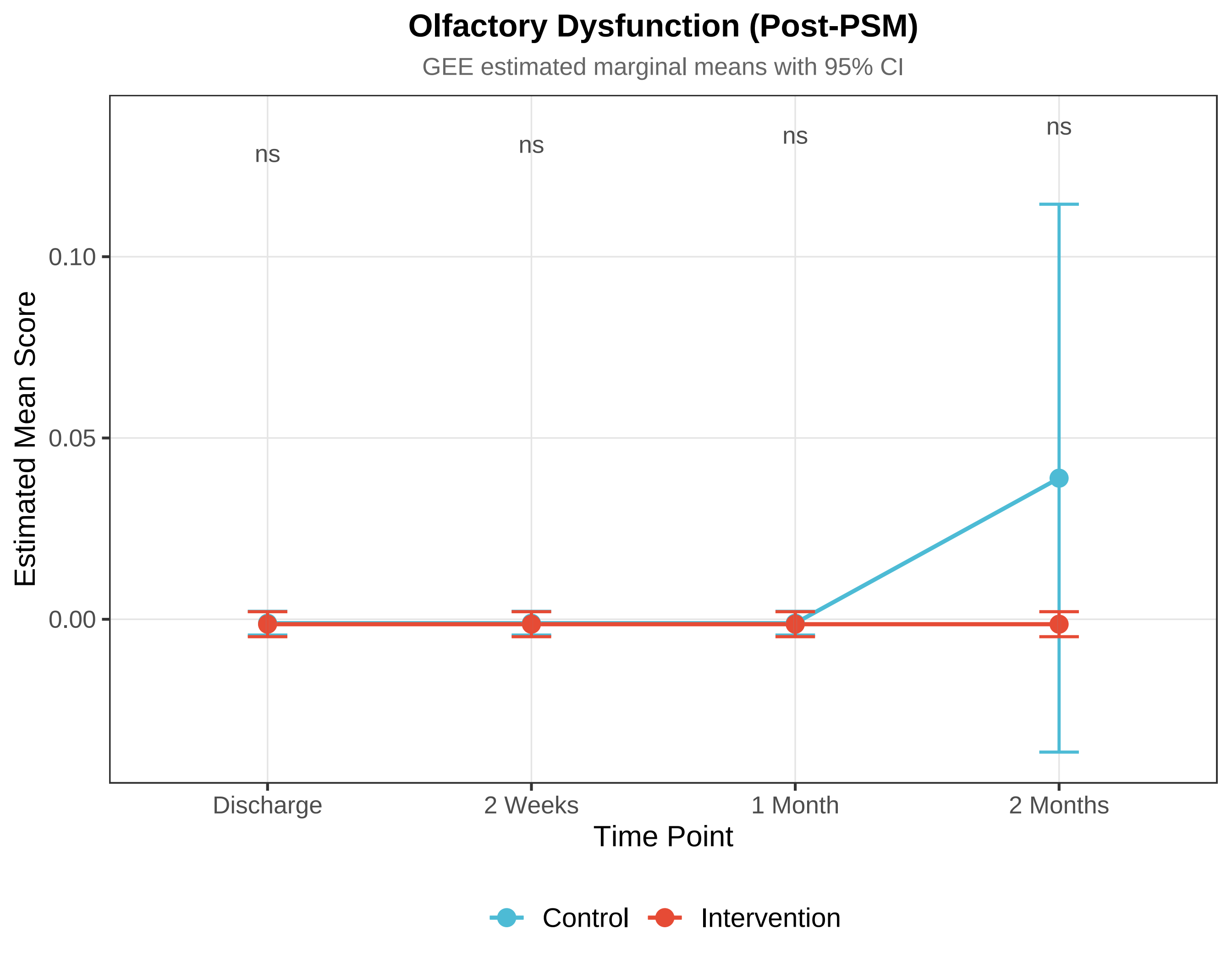
Olfactory Dysfunction (Post-PSM)
GEE estimated marginal means with 95% CI
ns
ns
ns
ns
0.10
0.05
0.00
Estimated Mean Score
Discharge
2 Weeks
1 Month
2 Months
Time Point
Control
Intervention
