# Supplementary material for: Association of nasal irrigation with reduced nasal symptoms after radioiodine ablation in differentiated thyroid cancer: a retrospective cohort study with propensity score matching
Source: Front Endocrinol (Lausanne). 2026 Sep 24;17:1897010. doi: 10.3389/fendo.2026.1897010 (PMC13645713; doi:10.3389/fendo.2026.1897010)
Supplement: Supplementary file 6 [file DataSheet2.pdf]

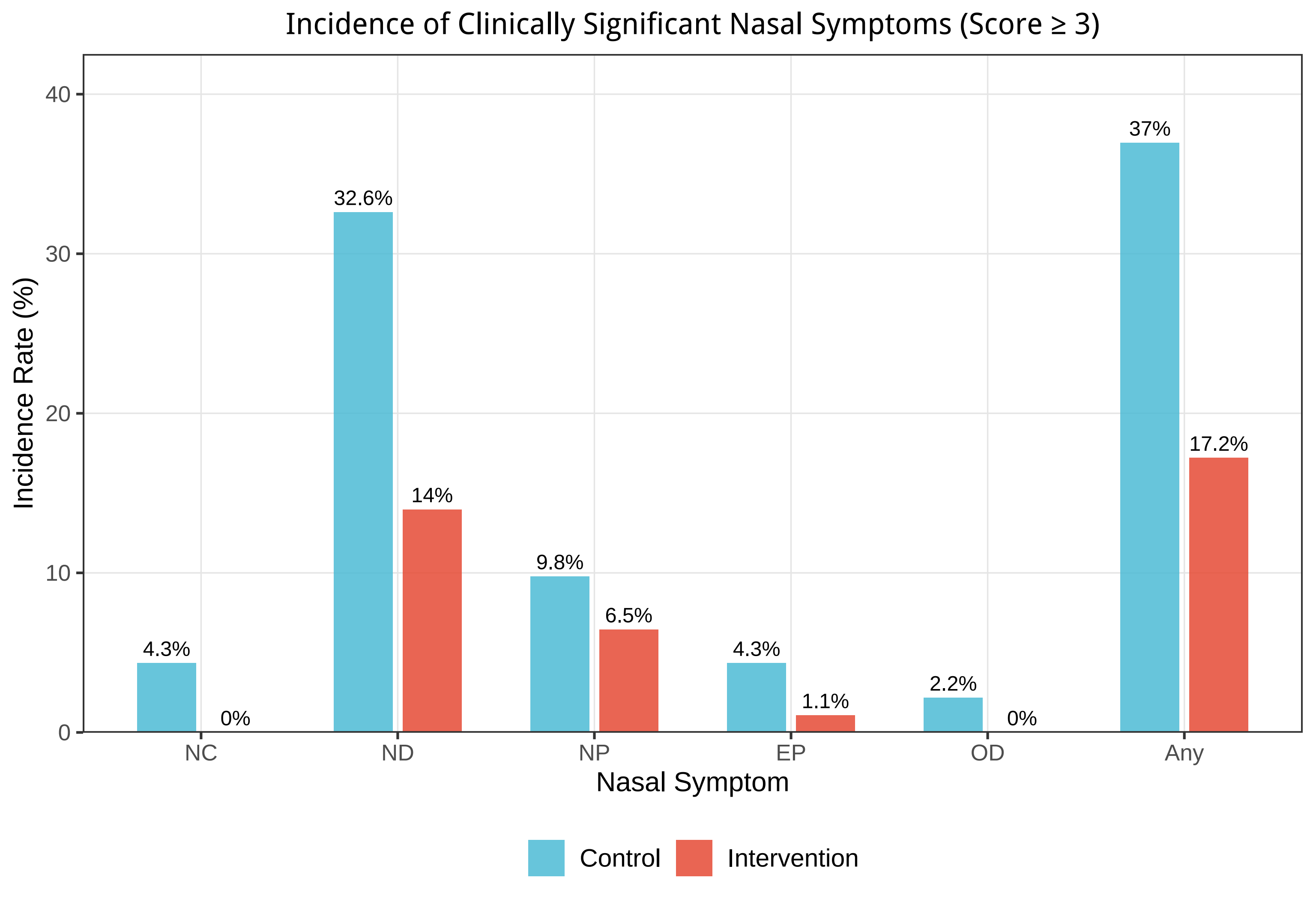
Incidence of Clinically Significant Nasal Symptoms (Score ≥ 3)
Incidence Rate (%)
40
30
20
10
0
4.3%
0%
32.6%
14%
9.8%
6.5%
4.3%
1.1%
2.2%
0%
37%
17.2%
NC
ND
NP
EP
OD
Any
Nasal Symptom
Control
Intervention
