# Supplementary material for: Association of nasal irrigation with reduced nasal symptoms after radioiodine ablation in differentiated thyroid cancer: a retrospective cohort study with propensity score matching
Source: Front Endocrinol (Lausanne). 2026 Sep 24;17:1897010. doi: 10.3389/fendo.2026.1897010 (PMC13645713; doi:10.3389/fendo.2026.1897010)
Supplement: Supplementary file 7 [file DataSheet3.pdf]

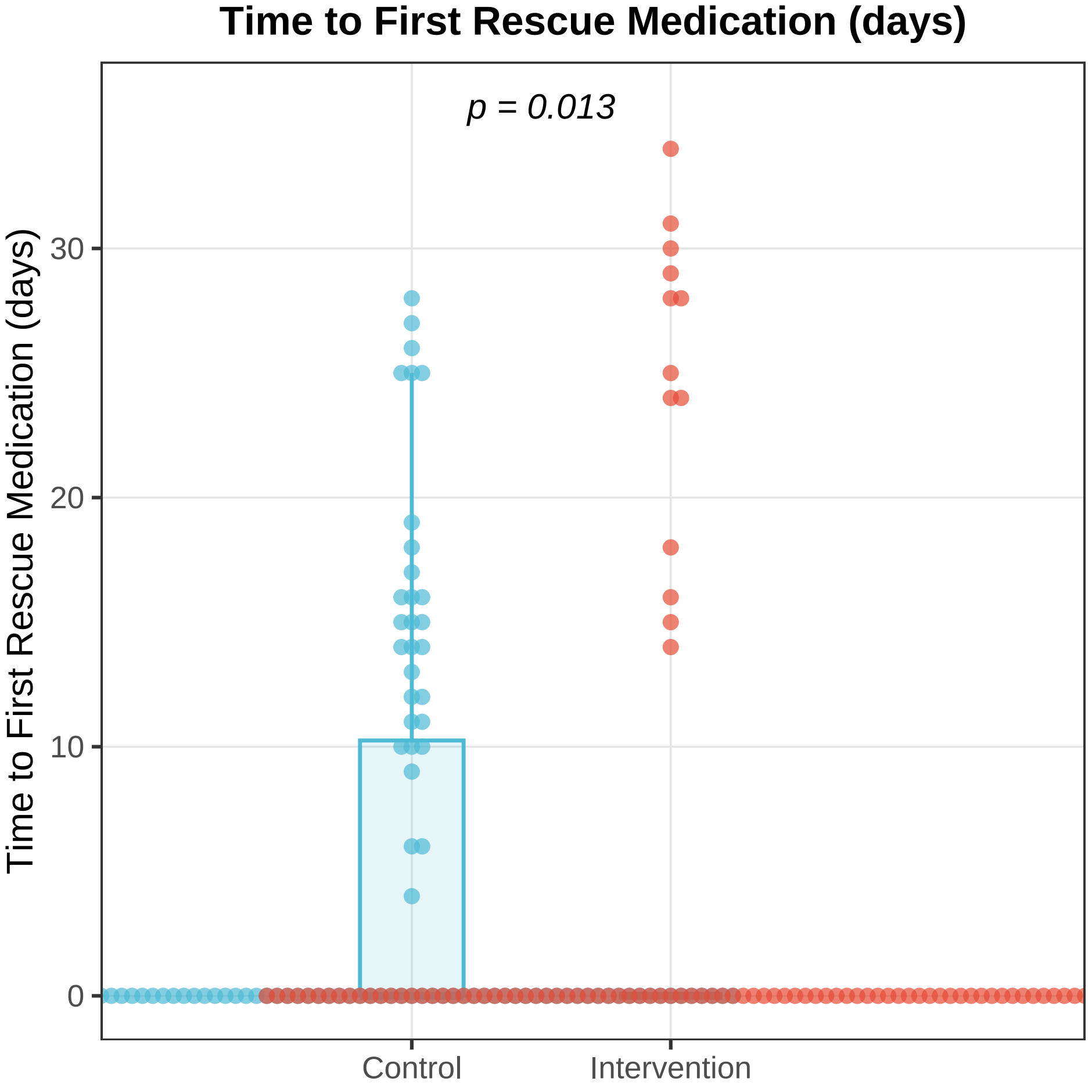

Time to First Rescue Medication (days)
p = 0.013
Time to First Rescue Medication (days)
30
20
10
0
Control
Intervention
