# Supplementary material for: Association of nasal irrigation with reduced nasal symptoms after radioiodine ablation in differentiated thyroid cancer: a retrospective cohort study with propensity score matching
Source: Front Endocrinol (Lausanne). 2026 Sep 24;17:1897010. doi: 10.3389/fendo.2026.1897010 (PMC13645713; doi:10.3389/fendo.2026.1897010)
Supplement: Supplementary file 13 [file DataSheet9.pdf]

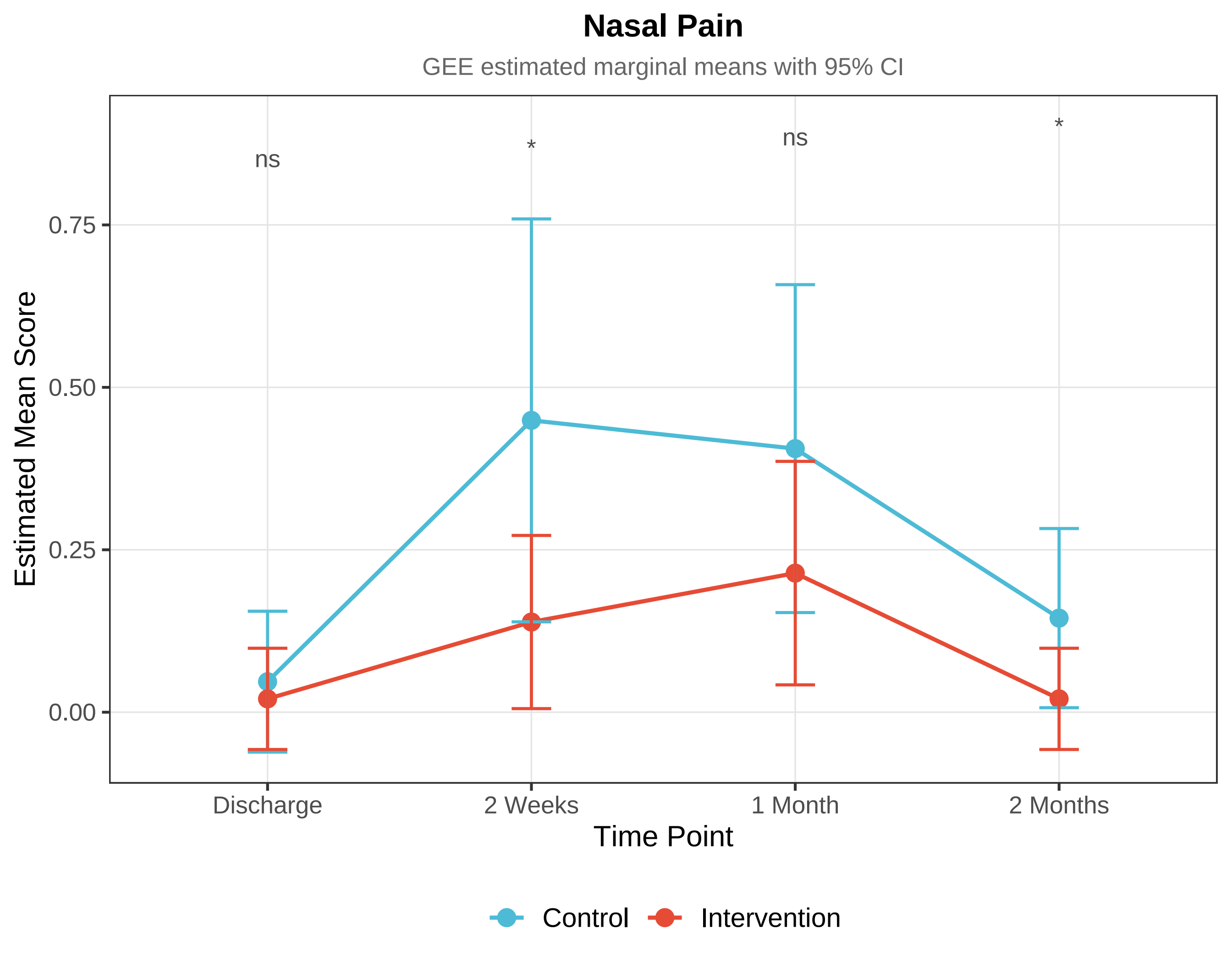

Nasal Pain
GEE estimated marginal means with 95% CI
Estimated Mean Score
0.00
0.25
0.50
0.75
ns
*
ns
*
Discharge
2 Weeks
1 Month
2 Months
Time Point
Control
Intervention
